# Clinical applications of multi-parametric CMR in myocarditis and systemic inflammatory diseases

**DOI:** 10.1007/s10554-017-1063-9

**Published:** 2017-01-27

**Authors:** Jakub Lagan, Matthias Schmitt, Christopher A. Miller

**Affiliations:** 1 North West Heart Centre, University Hospital of South Manchester, Manchester, UK; 2 Institute of Cardiovascular Sciences, Faculty of Medical & Human Sciences, University of Manchester, Manchester, M13 9NT UK

**Keywords:** Cardiac magnetic resonance, Myocarditis, Acute cardiac, Allograft rejection, Systemic lupus erythematosus, Rheumatoid arthritis, Sarcoidosis, Systemic sclerosis, T2 mapping, T1 mapping, Extracellular volume assessment, Late gadolinium enhancement

## Abstract

Cardiac magnetic resonance (CMR) has changed the management of suspected viral myocarditis by providing a ‘positive’ diagnostic test and has lead to new insights into myocardial involvement in systemic inflammatory conditions. In this review we analyse the use of CMR tissue characterisation techniques across the available studies including T2 weighted imaging, early gadolinium enhancement, late gadolinium enhancement, Lake Louise Criteria, T2 mapping, T1 mapping and extracellular volume assessment. We also discuss the use of multiparametric CMR in acute cardiac transplant rejection and a variety of inflammatory conditions such as sarcoidosis, systemic lupus erythrematous, rheumatoid arthritis and systemic sclerosis.

## Introduction

The World Health Organisation defines myocarditis as an inflammatory disease of the myocardium diagnosed by established histological, immunological and immunohistochemical criteria [1]. Myocardial involvement in presumed systemic viral infection is the most common aetiology, although it can result from a wide spectrum of infectious pathogens and non-infectious causes including systemic inflammatory conditions and toxins [2, 3].

Clinical presentation is often non-specific and heterogeneous, ranging from symptoms of chest pain, dyspnoea, fatigue or palpitations to brady- and tachy-arrhythmias, cardiogenic shock and sudden death [2]. Peripheral markers of inflammation (e.g. c-reactive protein) and myocardial injury (e.g. troponin) lack sensitivity and specificity, and viral serology is unhelpful [4–6]. Invasive endomyocardial biopsy (EMB) is recommended in specific scenarios, such as “New-onset heart failure of 2 weeks duration associated with a normal-sized or dilated left ventricle and hemodynamic compromise (IB)” and “New-onset heart failure of 2 weeks to 3 months duration associated with a dilated left ventricle and new ventricular arrhythmias, second- or third-degree heart block, or failure to respond to usual care within 1 to 2 weeks (1B)”, [7] however, it is associated with a risk of complications (1–2%) and due to sampling error, transiency of myocardial injury and variation in histology interpretation, it also lacks accuracy [2, 8–12]. In most centres it is rarely performed. As a result of these factors, the diagnosis of myocarditis is challenging, and has traditionally been made after other cardiac diseases have been excluded [2].

Over the past decade, cardiac magnetic resonance (CMR) has changed this paradigm. The unique ability of multiparametric CMR to characterise myocardial tissue, and thus potentially detect the myocardial oedema, increased blood flow and capillary leakage, necrosis and subsequent fibrosis that occurs in myocarditis, coupled with the ability of CMR to detect subtle regional or global contractile dysfunction, means that CMR is now often able to provide a positive diagnosis of myocarditis. Indeed, CMR has provided pathophysiological insight into the nature of the myocardial injury in myocarditis.

This review will describe the diagnostic utility of CMR parameters across a range of myocarditic aetiologies. In this context, it is important to recognise that the evaluation of CMR, or indeed any diagnostic test, in myocarditis is limited by the lack of a good reference standard. Histological validation is challenging and imperfect, as described. As a result, many studies use a clinical diagnosis of myocarditis as the reference, however this is inherently limited. In addition, heterogeneous study designs and patient populations (e.g. acute versus chronic myocardial inflammation, definition of control groups), and the nature of CMR (differing magnetic field strengths, imaging sequences, measured parameters) makes comparisons between studies difficult.

## Idiopathic (presumed viral) myocarditis

In North America and Europe, myocardial involvement in presumed systemic viral infection remains the most common aetiology of myocarditis [2, 13–18].

### T2 weighted imaging

T2 relaxation is directly proportional to tissue water content, and T2 weighted (T2w) imaging has been proposed to detect myocardial oedema [3, 19, 20].

Table 1 summarises studies that have evaluated the diagnostic performance of T2w imaging, including the sequences employed, the populations studied and the reference standards [21–35]. Most studies analyse T2w images using an oedema ratio (ER), defined as the ratio of myocardial to skeletal muscle signal intensity (SI), with values above a set value considered pathological. However, the threshold varies across studies (1.8–2.2), is usually determined retrospectively and the technique is hampered by potential coexistence of myositis and a lack of skeletal muscle in the field of view [21, 35]. A minority of studies have used qualitative assessment, although a lack of ‘healthy’ myocardium for comparison in the context of global myocarditis is a limitation [35].

**Table 1:** T2w Imaging

Study	Field strength	Sequence	Validation	Myocarditis (n)	Control (n)	Acute versus chronic cardiac inflammation	Control group	Test result	Sensitivity (%)	Specificity (%)	Diagnostic accuracy (%)	PPV (%)	NPV (%)
Laissy et al. Chest [21]	1 T	T2w sequence with long TR/TE	Clinical	20	7	Acute	Healthy participants	Qualitative	45	100	59	100	39
Abdel-Aty et al. J Am Col Cardiol [22]	1.5 T	T2w triple inversion recovery sequence	Clinical	25	23	Acute	Healthy participants	ER cut off 1.9	84	74	79	78	81
Gutberlet et al. Radiology [23]	1.5 T	T2w triple inversion recovery sequence	EMB	48	35	Chronic	Other diagnoses	ER cut off 2	67	69	68	74	60
Röttgen et al. Eur Radiol [24]	1.5 T	T2w triple inversion recovery sequence	EMB	82	49	Acute	No inflammation on EMB	ER cut off 2	58	57	58	73	41
Voigt et al. Eur Radiol [25]	1.5 T	T2w triple inversion recovery sequence	EMB	12	11	Chronic	No inflammation on EMB	ER cut off 1.8	92	82	87	85	90
Lurz et al. JACC Cardiovasc Imaging 26	1.5 T	T2w triple inversion recovery sequence	EMB	53	17	Acute	Other diagnoses	ER cut off 1.9	64	65	64	85	37
Lurz et al. JACC Cardiovasc Imaging [26]^a^	1.5 T	T2w triple inversion recovery sequence	EMB	30	32	Chronic	Other diagnoses	ER cut off 1.9	42	66	54	58	50
Chu et al. Int J Cardiovasc Im [27]	1.5 T	T2w triple inversion recovery sequence	Clinical	35	10	Acute	Healthy participants	Qualitative	69	100	76	100	48
Ferreira et al. JACC Cardiovasc Imaging [28]	1.5 T	Acquisition for cardiac unified T2 oedema sequence	Clinical	50	45	Acute	Healthy participants	ER cut off 2.2	67	55	61	78	42
Sramko et al. Am J Cardiol [29]	1.5 T	T2w dark blood sequence	EMB	15	27	Chronic	Idiopathic DCM	ER cut off 1.9	13	93	64	51	66
Ferreira et al. J Cardiovasc Magn Reson [30]	1.5 T	T2w triple inversion recovery sequence	Clinical	60	50	Acute	Healthy participants	ER cut off 2.0	48	86	65	81	58
Radunski et al. JACC Cardiovasc Imaging [31]	1.5 T	T2w triple inversion recovery sequence	Clinical	104	21	Mostly Acute	Healthy Participants	ER cut off 2.2	76	42	70	84	30
Luetkens et al. Radiology [32]	3 T	T2w triple inversion recovery sequence	Clinical	24	42	Acute	Healthy Participants	ER cut off 2.09	79	61	68	58	82
Schwab et al. Rofo [33]	1.5 T	T2w triple inversion recovery sequence	Clinical	43	35	Acute	Healthy participants	Qualitative	56	100	76	100	65
Hinojar et al. JACC Cardiovasc Imaging [34]	1.5 T / 3 T	T2w triple inversion recovery sequence	Clinical	61	40	Acute	Healthy participants	Qualitative/ER	56	94	71	95	55
Luetkens et al. Eur H J Cardiovasc im [35]	1.5 T	T2w triple inversion recovery sequence	Clinical	34	50	Acute	Healthy participants	ER cut off 1.9	50	94	76	85	73
Pooled data				696	494				62	76	67	78	58
Chronic inflammation only									55	76	65	69	63
Acute inflammation only									63	76	68	80	57
Healthy participants as control									64	79	70	81	61
Other diagnoses as controls									58	69	63	73	54

*DCM* dilated cardiomyopathy, *ER* oedema ratio, *EMB* endomyocardial biopsy, *NPV* negative predictive value, *PPV* positive predictive value, *TE* echo time, *TR* repeat time; *T2w* T2 weighted

^a^One study examining two groups of patients with acute and chronic cardiac inflammation

The pooled weighted sensitivity, specificity and diagnostic accuracy of T2w for diagnosing acute myocarditis are 63, 76 and 68% respectively.

In the largest study (104 patients), in which a clinical diagnosis of myocarditis was used as the reference standard, Radunski et al. reported a modest diagnostic accuracy (70%) [31]. Median interval between symptom onset and scan was 2 weeks, however the interquartile range was up to 7 weeks, by which time patients may have been in the convalescent stage. Indeed, the effect of delayed scan timing on T2w imaging sensitivity was investigated by Monney et al [36] and Hinojar et al [34], who found a higher prevalence of abnormal signal on T2w images when scanning within 2 weeks of symptom onset (81 and 56% respectively) compared to scanning performed later (11% at 39 days [36] and 12% at 6 months [34]). Other studies comparing acute and convalescent imaging have also shown that high T2 signal is a transient feature of inflammatory response [36–40]. In addition, abnormalities detectable on T2w imaging appear to vary according to clinical presentation, with a higher prevalence in the context of infarction-like symptoms (81% sensitivity) and much lower in the setting of heart failure or arrhythmias (sensitivity 28 and 27% respectively) [41].

### Early gadolinium enhancement

Early gadolinium enhancement (EGE) exploits the phenomenon of regional vasodilatation, increased blood flow and capillary leakage present in an inflammatory process which results in increased contrast retention in the early washout period [3].

Table 2 summarises studies that have evaluated the diagnostic performance of EGE imaging [3, 21–27, 29, 31–33, 35, 42]. Analysis of EGE images is performed using Myocardial Signal Enhancement, defined as myocardial SI post-contrast minus myocardial SI pre- contrast divided by myocardial SI pre- contrast, with values above 45–56% considered pathological [21, 29, 31], or more commonly, the global relative enhancement (gRE),[42] which is calculated as myocardial signal enhancement divided by skeletal muscle signal enhancement. Most studies use a gRE value of 4.0 as the threshold between healthy and abnormal myocardium [22–27, 32]. Such analyses have similar disadvantages to the ER.

**Table 2:** Early gadolinium enhancement

Study	Field strength	Sequence	Validation	Myocarditis (n)	Control (n)	Acute versus chronic cardiac inflammation	Control group	Test result	Sensitivity (%)	Specificity (%)	Diagnostic accuracy (%)	PPV (%)	NPV (%)
Friedrich et al. Circulation [3, 42]	1 T	T1w spin echo sequence	Clinical	19	18	Acute	Healthy Participants	gRE	84	89	86	89	84
Laissy et al. Chest [3, 21]	1 T	T1w sequence with short TR/TE	Clinical	20	7	Acute	Healthy Participants	MSE cut off 45%	85	100	89	100	70
Abdel-Aty et al. J Am Col Cardiol [22]	1.5 T	T1w spin echo sequence	Clinical	25	23	Acute	Healthy Participants	gRE cut off 4.0	80	68	75	74	75
Gutberlet et al. Radiology [23]	1.5 T	T1w fast spin echo sequence	EMB	48	35	Chronic	Other diagnoses	gRE cut off 4.0	63	86	73	86	63
Röttgen et al. Eur Radiol [24]	1.5 T	T1w fast spin echo sequence	EMB	82	49	Acute	No inflammation on EMB	gRE cut off 4.0	49	74	57	78	43
Voigt et al. Eur Radiol [25]	1.5 T	T1w spin echo sequence	EMB	12	11	Chronic	No inflammation on EMB	gRE cut off 4.0	58	64	61	64	58
Lurz et al. JACC Cardiovasc Imaging [26]^a^	1.5 T	T1w fast spin echo sequence	EMB	53	17	Acute	Other diagnoses	gRE cut off 4.0	76	53	70	83	41
Lurz et al. JACC Cardiovasc Imaging [26]^a^	1.5 T	T1w fast spin echo sequence	EMB	30	32	Chronic	Other diagnoses	gRE cut off 4.0	73	21	48	51	40
Chu et al. Int J Cardiovasc I [27]	1.5 T	T1w turbo spin echo sequence	Clinical	35	10	Acute	Healthy Participants	gRE cut off 4.0	63	90	69	96	41
Sramko et al. Am J Cardiol [29]	1.5 T	T1w turbo flash sequence	EMB	15	27	Chronic	Idiopathic DCM	MSE cut off 45%	40	96	76	85	74
Radunski et al. JACC Cardiovasc Imaging [31]	1.5 T	T1w spin echo sequence	Clinical	104	21	Mostly Acute	Healthy Participants	MSE cut off 56%	63	71	59	91	31
Luetkens et al. Radiology [32]	3 T	T1w fast spin echo sequence	Clinical	24	42	Acute	Healthy Participants	gRE cut off 4.0	83	42	60	53	77
Schwab et al. Rofo [33]	1.5 T	T1w fast spin echo sequence	Clinical	43	35	Acute	Healthy Participants	Qualitative assessment	51	94	71	92	61
Luetkens et al. Eur H J Cardiovasc im [35]	1.5 T	T1w fast spin echo sequence	Clinical	34	50	Acute	Healthy Participants	gRE cut off 1.95	77	62	67	58	80
Pooled data				544	377				65	69	67	75	58
Chronic inflammation only									62	66	64	65	64
Acute inflammation only									66	70	67	78	56
Healthy participants as control									69	70	69	77	60
Other diagnoses as controls									61	67	63	72	55

*DCM* dilated cardiomyopathy, *EMB* endomyocardial biopsy, *gRE* – global relative enhancement, *MSE* myocardial signal enhancement, *NPV* negative predictive value, *PPV* positive predictive value, *TE* echo time, *TR* repeat time, *T1w* T1 weighted

^a^One study examining two groups of patients with acute and chronic cardiac inflammation

The pooled weighted sensitivity, specificity and diagnostic accuracy of EGE for diagnosing acute myocarditis are 66, 70 and 67% respectively, with a wide range of diagnostic performances reported for both myocardial signal enhancement and gRE analysis techniques. Interestingly, Bohnen et al. found no statistical difference in gRE between heart failure patients with histologically confirmed inflammation and those without [43].

Friedrich et al [42, 44] found the pattern of signal enhancement was localised within first week but subsequently became more diffuse. By day 14, gRE values stopped being significantly higher in the myocarditis group compared to the control group. Studies comparing EGE in acute and convalescent phases show a significant drop in gRE, from 4.1–8.5 during acute presentation to 2.4–4.4 at follow up (performed 3–28 months later) [37–40].

### Late gadolinium enhancement

**Fig. 1:**
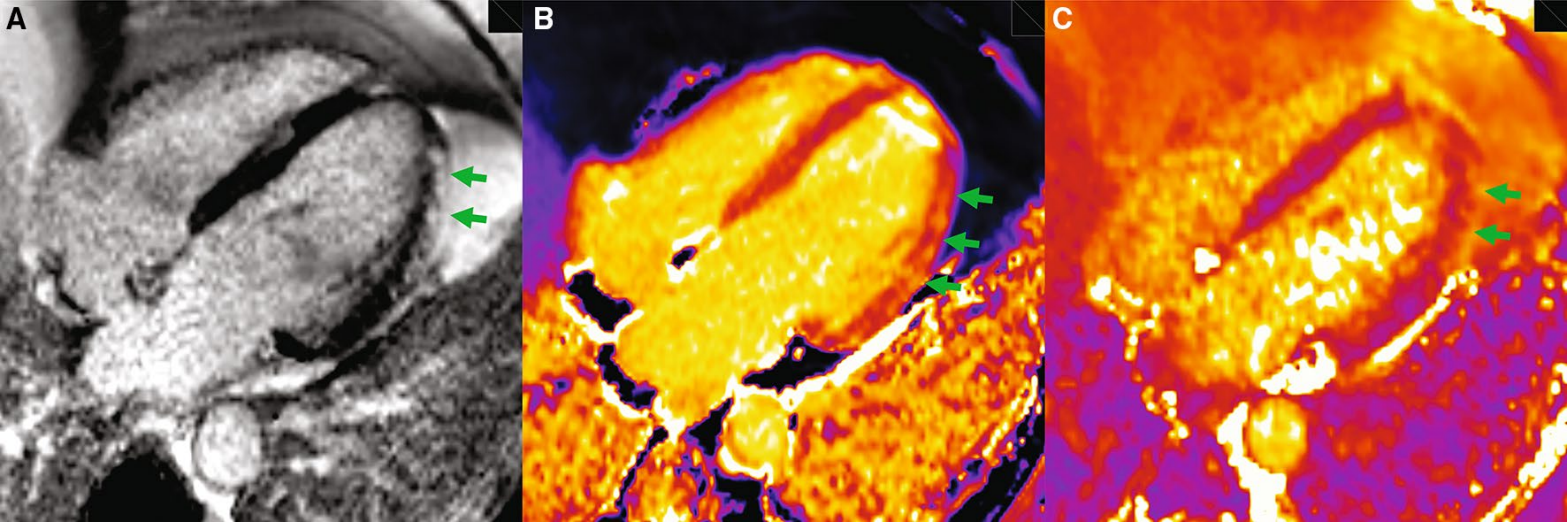
Patient with acute viral myocarditis. **a** Late enhancement imaging. Epicardial and mid-wall late enhancement (*green arrows*) in mid anterolateral and apical lateral segments. **b** T1 mapping, MOLLI sequence. Elevated T1 values in mid-wall and epicardial portion of basal—mid anterolateral and apical lateral segments (*green arrows*; T1 values in anterolateral wall: 1152 ms, T1 values in basal inferoseptum: 1031 ms). **c** T2 mapping, T2-prepared SFFP sequence. Elevated T2 values in epicardial portion of mid anterolateral and apical lateral segments (*green arrows*; T2 values in mid anterolateral segment: 66 ms, T2 values in basal inferoseptum: 47 ms)

Late gadolinium enhancement (LGE) was originally thought to demonstrate irreversible myocardial injury only, however several studies have demonstrated a temporal change in the extent of LGE in myocarditis, with LGE volume seen to decrease significantly over time (follow up scans performed between 1 and 18 months) [3, 36–38, 40, 45]. Histological correlation has shown LGE is associated with active inflammation, with the extent of LGE corresponding to the severity of the inflammatory histopathological findings [45, 46]. It is likely that LGE in acute myocarditis represents both reversible and irreversible myocardial injury, but in the chronic phase represents residual focal fibrosis. See Fig. 1a for a representative example.

Table 3 summarises studies that have evaluated the diagnostic performance of LGE imaging [22–27, 29–35, 45, 47]. The pooled weighted sensitivity, specificity and diagnostic accuracy of LGE for diagnosing acute myocarditis are 65, 95 and 75% respectively. The prevalence of LGE varies considerably across studies (27–95%), likely reflecting the heterogeneity of the populations studied and the timing of CMR [22–27, 29–36, 40, 45, 47–51].

**Table 3:** Late gadolinium enhancement

Study	Field strength	Sequence	Validation	Myocarditis (n)	Control (n)	Acute versus chronic cardiac inflammation	Control group	Sensitivity (%)	Specificity (%)	Diagn accuracy (%)	PPV (%)	NPV (%)
Abdel-Aty et al. J Am Col Cardiol [22]	1.5 T	Inversion-recovery gradient echo sequence	Clinical	25	23	Acute	Healthy participants	44	100	71	78	62
Mahrholdt et al. Circulation [45]	1.5 T	Inversion-recovery gradient echo sequence	EMB	102	26	Acute	No inflammation on EMB	87	96	89	99	65
Gutberlet et al. Radiology [23]	1.5 T	Inversion-recovery gradient echo sequence	EMB	48	35	Chronic	Other diagnoses	27	80	49	65	44
Yilmaz et al. Heart [47]	1.5 T	Inversion-recovery gradient echo sequence	EMB	48	23	Acute	No inflammation on EMB	35	83	51	81	38
Röttgen et al. Eur Radiol [24]	1.5 T	Inversion-recovery gradient echo sequence	EMB	82	49	Acute	No inflammation on EMB	31	88	52	84	39
Voigt et al. Eur Radiol [25]	1.5 T	Inversion-recovery gradient echo sequence	EMB	12	11	Chronic	No inflammation on EMB	58	45	52	53	50
Lurz et al. JACC Cardiovasc Imaging [26]^a^	1.5 T	Inversion-recovery turbo gradient echo sequence	EMB	53	17	Acute	Other diagnoses	74	65	72	87	44
Lurz et al. JACC Cardiovasc Imaging [26]^a^	1.5 T	Inversion-recovery turbo gradient echo sequence	EMB	30	32	Chronic	Other diagnoses	61	35	48	51	44
Chu et al. Int J Cardiovasc Im [27]	1.5 T	Inversion-recovery gradient echo sequence	Clinical	35	10	Acute	Healthy Participants	77	60	73	87	43
Sramko et al. Am J Cardiol [29]	1.5 T	Phase-sensitive inversion-recovery sequence	EMB	15	27	Chronic	Idiopathic DCM	87	44	59	46	86
Ferreira et al. J Cardiovasc Magn Reson [30]	1.5 T	Phase-sensitive inversion-recovery sequence	Clinical	60	50	Acute	Healthy Participants	72	97	83	98	67
Radunski et al. JACC Cardiovasc Imaging [31]	1.5 T	Phase-sensitive inversion-recovery sequence	Clinical	104	21	Mostly Acute	Healthy Participants	61	100	68	100	34
Luetkens et al. Radiology [32]	3 T	Inversion-recovery gradient echo sequence	Clinical	24	42	Acute	Healthy Participants	75	100	91	100	88
Schwab et al. Rofo [33]	1.5 T	Inversion-recovery turbo gradient echo sequence	Clinical	43	35	Acute	Healthy Participants	86	100	92	100	85
Hinojar et al. JACC Cardiovasc Imaging [34]	1.5 T / 3 T	Inversion-recovery sequence	Clinical	61	40	Acute	Healthy Participants	72	100	83	100	79
Luetkens et al. Eur H J Cardiovasc im [35]	1.5 T	Inversion-recovery gradient echo sequence	Clinical	34	50	Acute	Healthy Participants	74	100	89	100	85
Pooled data				776	491			63	85	72	87	59
Chronic inflammation only								49	53	51	51	51
Acute inflammation only								65	95	75	96	59
Healthy participants as control								70	98	81	98	69
Other diagnoses as controls								57	70	62	77	48

^a^One study examining two groups of patients with acute and chronic cardiac inflammation

*DCM* dilated cardiomyopathy;* EMB* endomyocardial biopsy;* NPV* negative predictive value;* PPV* positive predictive value

While a number of studies have shown that LGE can involve any region of the LV (or the right ventricle), Mahrholdt et al.[46] in seminal work, showed LGE is most commonly located in the lateral LV, typically originating from the epicardial quartile of the LV wall. Transmural lateral wall LGE, possibly reflecting very florid disease, is reported in a minority [22, 23, 26, 27, 30, 33, 36, 40, 45, 47, 49–53]. The distribution of LGE may be associated with the infecting pathogen, with parvovirus B19 found to be association with sub-epicardial lateral wall LGE, whereas human herpes virus 6 is associated with mid wall septal LGE [45].

LGE imaging requires the presence of ‘normal’ myocardium as a reference, thus may not be sensitive to diffuse disease [54].

### Lake Louise criteria

In an effort to increase the diagnostic performance of CMR, the three tissue characterisation techniques discussed above (T2w imaging, EGE and LGE) were combined to form the Lake Louise Criteria (LLC). In the setting of clinically suspected myocarditis, abnormal findings on two of the three techniques were determined to be consistent with myocardial inflammation [3].

Table 4 summarises studies that have evaluated the diagnostic performance of the LLC [22, 23, 25–27, 31–33, 35]. The pooled weighted sensitivity, specificity and diagnostic accuracy of the LLC for diagnosing acute myocarditis are 80, 87 and 83% respectively, and as such the LLC demonstrate a better overall diagnostic performance than any of the individual CMR parameters. Similar to T2w imaging, LLC appears to have better diagnostic performance in “infarct-like” presentation (sensitivity of 80%) compared to heart failure or arrhythmias (sensitivity 57 and 40% respectively) [41].

**Table 4:** Lake Louise criteria

Study	Field strength	Validation	Myocarditis (n)	Control (n)	Acute versus chronic cardiac inflammation	Control group	CMR sequences	Sensitivity (%)	Specificity (%)	Diagnostic accuracy (%)	PPV (%)	NPV (%)
Abdel-Aty et al. J Am Col Cardiol [3, 22]	1.5 T	Clinical	25	23	Acute	Healthy Participants	ER, gRE, LGE	76	96	86	95	79
Gutberlet et al. Radiology [23]	1.5 T	EMB	48	35	Chronic	Other diagnoses	ER, gRE, LGE	63	89	74	88	63
Voigt et al. Eur Radiol [25]	1.5 T	EMB	12	11	Chronic	No inflammation on EMB	ER, gRE, LGE	75	73	74	75	73
Lurz et al. JACC Cardiovasc Imaging [26]^a^	1.5 T	EMB	53	17	Acute	Other diagnoses	ER, gRE, LGE	81	71	79	90	55
Lurz et al. JACC Cardiovasc Imaging [26]^a^	1.5 T	EMB	30	32	Chronic	Other diagnoses	ER, gRE, LGE	63	40	51	53	50
Chu et al. Int J Cardiovasc Im [27]	1.5 T	Clinical	35	10	Acute	Healthy Participants	Qualitative T2w assessment, gRE, LGE	77	90	80	96%	53
Radunski et al. JACC Cardiovasc Imaging [31]	1.5 T	Clinical	104	21	Mostly Acute	Healthy Participants	ER, MSE, LGE	84	57	79	90	41
Luetkens et al. Radiology [32]	3 T	Clinical	24	42	Acute	Healthy Participants	ER, gRE, LGE	92	80	85	79	92
Schwab et al. Rofo [33]	1.5 T	Clinical	43	35	Acute	Healthy Participants	Qualitative T2w assessment, qualitative EGE assessment, LGE	67	100	82	100	72
Luetkens et al. Eur H J Cardiovasc im [35]	1.5 T	Clinical	34	50	Acute	Healthy Participants	ER, gRE, LGE	82	98	92	97	89
Pooled data			408	276				77	81	79	86	70
Chronic inflammation only								65	67	66	69	62
Acute inflammation only								80	87	83	91	73
Healthy participants as control								80	89	84	91	75
Other diagnoses as controls								71	67	69	77	60

*DCM* dilated cardiomyopathy, *ER* oedema ratio, *EMB* endomyocardial biopsy, *gRE* global relative enhancement, *MSE* myocardial signal enhancement, *NPV* negative predictive value, *PPV* positive predictive value, *T1w* T1 weighted, *T2w* T2 weighted

^a^One study examining two groups of patients with acute and chronic cardiac inflammation

### Parametric mapping

In recent years, parametric mapping, which allows direct quantification of myocardial tissue magnetic parameters (primarily T1 and T2) has been increasingly applied in myocarditis. (Similar to T2, T1 relaxation times are sensitive to changes in myocardial water content and have been proposed to detect myocardial oedema). As well as being associated with potentially less observer variability, less artefact and allowing global myocardial assessment, native T1 and T2 mapping offer the significant advantage of not requiring contrast agent administration. See Fig. 1b, c for representative examples.

Table 5 summarises the studies that have evaluated the diagnostic performance of T2 and T1 mapping. The pooled weighted sensitivity, specificity and diagnostic accuracy of T2 mapping for diagnosing acute myocarditis are 70, 91 and 79% respectively [31, 35, 43, 51, 55]. The pooled weighted sensitivity, specificity and diagnostic accuracy of T1 mapping are 82, 91 and 86% [30–32, 34, 35]. Thus the diagnostic performance of T2 mapping is comparable to that of the LLC, while the performance of T1 mapping may be superior.

**Table 5:** Parametric mapping

Study	Field strength	Sequence	Validation	Myocarditis (n)	Control (n)	Acute versus chronic cardiac inflammation	Control group	Test result	Sensitivity (%)	Specificity (%)	Diagnostic accuracy (%)	PPV (%)	NPV (%)
**T2 mapping**													
Thavendiranathan et al. Circ Cardiovasc Imaging [51]	1.5 T	T2p-SFFP	Clinical	20	30	Acute	Healthy participants	T2 cut off 59 ms	94	97	96	95	96
Radunski et al. JACC Cardiovasc Imaging [31]	1.5 T	T2 multiecho sequence	Clinical	87	21	Mostly Acute	Healthy participants	T2 cut off 61 ms	57	89	63	95	35
Bohnen et al. Circ Cardiovasc Imaging [43]	1.5 T	Hybrid gradient and spin-echo multiecho sequence	EMB	16	15	Chronic	No inflammation on EMB	T2 cut off 60 ms	94	60	78	71	90
Baessler et al. J Cardiovasc Magn Reson [55]	1.5 T	GraSE	LLC	31	30	Acute	Healthy participants	max T2 68 ms/madSD 0.22	81	83	82	83	81
Luetkens et al. Eur H J Cardiovasc Im [35]	1.5 T	GraSE	Clinical	34	50	Acute	Healthy participants	T2 cut off 59.9 ms	79	92	87	87	87
Pooled data				188	146				72	87	79	88	71
Chronic inflammation only									94	60	78	71	90
Acute inflammation only									70	91	79	91	70
Healthy participants as control									70	91	79	91	70
Other diagnoses as controls									94	60	78	71	90
**T1 mapping**													
Ferreira et al. J Cardiovasc Magn Reson [30]	1.5 T	ShMOLLI	Clinical	60	50	Acute	Healthy participants	T1 cut off 990 ms	90	88	89	90	88
Luetkens et al. Radiology [32]	3 T	MOLLI	Clinical	24	42	Acute	Healthy participants	T1 cut off 1140 ms	92	91	91	85	95
Radunski et al. JACC Cardiovasc Imaging [31]	1.5 T	MOLLI	Clinical	104	21	Mostly Acute	Healthy participants	T1 cut off 1074 ms	64	90	68	97	34
Hinojar et al. JACC Cardiovasc Imaging [34]	1.5 T / 3 T	MOLLI	Clinical	61	40	Acute	Healthy participants	T1 cut off 992 ms on 1.5 T, 1098 ms on 3 T	98	100	99	100	99
Luetkens et al. Eur H J Cardiovasc im [35]^a^	1.5 T	MOLLI	Clinical	34	50	Acute	Healthy participants	T1 cut off 1000 ms	85	96	92	94	90
Luetkens et al. Eur H J Cardiovasc im [35]^a^	1.5 T	ShMOLLI	Clinical	34	50	Acute	Healthy participants	T1 cut off 852 ms	88	84	86	79	91
Pooled data				317	253				82	91	86	92	81
**ECV**													
Luetkens et al. Radiology [32]	3 T	MOLLI	Clinical	24	42	Acute	Healthy participants	ECV cut off 26%	67	81	76	67	81
Radunski et al. JACC Cardiovasc Imaging [31]	1.5 T	MOLLI	Clinical	104	21	Mostly Acute	Healthy participants	ECV cut off 29%	73	90	76	97	40
Luetkens et al. Eur H J Cardiovasc im [35]^a^	1.5 T	MOLLI	Clinical	34	50	Acute	Healthy participants	ECV cut off 29%	70	76	74	67	79
Luetkens et al. Eur H J Cardiovasc im [35]^a^	1.5 T	ShMOLLI	Clinical	34	50	Acute	Healthy participants	ECV cut off 30%	57	92	78	83	75
Pooled data				196	163				69	84	76	84	69

*DCM* dilated cardiomyopathy;* EMB* endomyocardial biopsy;* GraSE* Gradient spin echo T2 sequence;* LLC* Lake Louise Criteria;* MOLLI* Modified Look-Locker inversion recovery sequence;* NPV* negative predictive value;* PPV* positive predictive value;* ShMOLLI* Shortened modified Look-Locker inversion recovery sequence;* T2p-SFFP* T2 prepared steady-state free precession sequence

^a^Two seprate T1 mapping sequences employed in one study: MOLLI and ShMOLLI

Luetkens et al. compared the diagnostic performance of CMR parameters in two studies, albeit in relatively small populations (24 and 34 patients with myocarditis respectively), and demonstrated similar findings. In the first study, which did not include T2 mapping [32], native T1 mapping was associated with the highest diagnostic performance (area under the curve, AUC 0.94), followed by LGE (AUC 0.9), LLC (AUC 0.86), ER (AUC 0.79) and gRE (AUC 0.63). In the second study, which included T2 mapping,[35], the performance of native T1 mapping (AUC 0.92–0.95) and T2 mapping (AUC 0.92) was very similar. Combining T1 mapping with LGE (diagnostic accuracy 91–96%) [30, 32, 34, 35] or T2 mapping and LGE (diagnostic accuracy 96%) [35] may improve diagnostic performance further.

Nevertheless, there are a number of areas which require further investigation. Only one study has compared T1 and T2 mapping with histological findings in myocarditis. Relaxation time thresholds for diagnosing myocarditis have generally been determined retrospectively. T1 relaxation time diagnostic thresholds vary considerably between studies (852–1074 ms at 1.5 T). T2 relaxation time diagnostic thresholds are generally much more consistent (approximately 60 ms), however they overlap considerably with published normal ranges (up to 65 ms) [51, 56–66]. A prospective, multicentre, multivendor trial with predetermined diagnostic thresholds is required to determine the clinical diagnostic utility of mapping with quantitative analysis before this technique can enter clinical practice.

Other noteworthy findings include those of Hinojar et al. who showed elevated T1 values (compared to healthy controls) persisted for up to 4–8 months post initial presentation [34]. Bohnen et al. found no difference in T1 values in patients with heart failure and histologically confirmed inflammation compared to patients with heart failure and no evidence of inflammation on histology [43]. This may reflect the fact that native T1 is determined by a number of factors other than inflammation (e.g. fibrosis).

Only three studies have examined the diagnostic utility of ECV in myocarditis, with varying results (Table 5) [31, 32, 35].

## Acute cardiac allograft rejection

Acute cardiac allograft rejection (ACAR) is a leading cause of death in the first year post heart transplant, however clinical features are unreliable. Routine screening is therefore performed in order to detect ACAR and hence augment immunosuppressive therapy, at an earlier stage, with the aim of preventing progression to more severe disease [67, 68]. Histological analysis of myocardial tissue obtained at EMB remains the gold standard for ACAR surveillance however it is associated with a number of limitations. CMR is a potentially attractive screening modality.

In one of the largest human studies, which included 68 patients undergoing 123 CMR scans, T2 relaxation time was significantly higher in grade 2 ACAR (57 ± 5 ms) compared with grade 0 or 1 (50 ± 5 ms and 51 ± 8 ms, respectively); and in grade 3 (65 ± 8 ms) compared with grade 2 [69]. A T2 relaxation time of ≥56 ms, determined retrospectively, had a high NPV (97%) for detecting significant ACAR (≥grade 2). More recently in a study of approximately 50 patients undergoing 68 CMR scans, Usman et al. found myocardial T2 was significantly higher in the ACAR group (including 4 cases of >grade 2R ACAR, two cases of antibody-mediated rejection and two cases where ACAR treatment was started on the basis of high clinical suspicion alone) compared to the non-ACAR group [70]. A T2 of 56.4 ms yielded a sensitivity and specificity of 86.5 and 94.6% respectively. However, both studies specifically selected patients who were known to have/suspected of having ACAR. Furthermore, patients were a scanned at a substantial time post-transplant (Marie et al. up to 6 years, Usman et al. up to 2 years), thus missing the window in which early detection of ACAR is thought to be most useful, indeed the benefit of routine screening later than one year post-transplant is subject to debate.

In a study of 22 patients undergoing 88 CMR scans over the first 5 months post-transplant, Miller et al. found myocardial T1 and T2 were not significantly higher in grade 2R ACAR compared to grades 0R-1R [71]. However the study did demonstrate significant improvements in markers of LV structure and contractility, native T1, T2 and ECV and microvascular function over the period studied, providing insight into the myocardial injury associated with transplantation, and its recovery.

It may be that CMR parameters become more useful for detecting ACAR as time from transplantation increases and the transplant-related myocardial injury subsides. The paradox however is that while non-invasive approaches to ACAR surveillance may become more discriminatory as time from transplantation increases, the benefit of the early detection of ACAR diminishes [71].

## Sarcoidosis

Sarcoidosis is a multi-organ systemic inflammatory disorder characterized by the formation of non-caseating granulomas [72]. Autopsy studies suggest cardiac sarcoidosis is a major cause for sarcoid-related mortality, however pre-mortem diagnosis of cardiac sarcoid is challenging [72, 73]. Endomyocardial biopsy and clinical diagnostic criteria [74] are limited [75].

Smedema et al. [76] found LGE in all patients (n = 12) meeting clinical criteria for cardiac sarcoid, and in a further 17% who did not meet the criteria. Patel et al. [77] showed CMR identified twice as many patients (n = 21) with evidence of myocardial involvement as clinical evaluation, which included 12-lead ECG and at last one non-CMR cardiac investigation (echocardiography, radionuclide scintigraphy or cardiac catheterisation).

Regional and mural LGE distribution in cardiac sarcoid is markedly heterogeneous. LGE has been demonstrated in all LV and RV regions, albeit with some predilection to basal septal regions [76–80]. Subendocardial, mid wall, epicardial and transmural patterns have been described [76–80]. Using T2 mapping, Crouser et al. [81] found significantly higher myocardial T2 values amongst 50 consecutive patients investigated for cardiac sarcoid compared to healthy controls. T2 cut off of 59 ms achieved sensitivity of 54% and specificity of 100%.

^18^F-fluoro-2-deoxyglucose positron emission tomography (^18^F-FDG PET; a marker of active inflammation) studies have provided insight into the CMR findings [82, 83]. T2w signal and LGE have been demonstrated to correspond to regions taking up ^18^F-FDG, with reduced uptake following corticosteroids, indicating active inflammation. However, LGE is also found in regions without ^18^F-FDG uptake, indicating fibrotic lesions. Thus T2w signal may reflect active inflammation, whereas LGE may reflect either active inflammation or fibrosis.

The presence of LGE is associated with a higher rate of sudden cardiac death (SCD) and ventricular tachyarrhythmia, although this requires further assessment in larger studies [77–79, 84].

## Systemic lupus erythematous

Systemic lupus erythematous (SLE) is a multisystem inflammatory disorder [85].

Cardiovascular involvement represents a significant cause of morbidity and mortality [86]. SLE associated myocarditis was shown to shorten the survival and is more common amongst patients with higher disease activity [87]. There is also a discrepancy between the number of myocarditis cases detected on autopsy and clinical diagnoses, suggesting common subclinical cardiac involvement [88, 89]. There is considerable interest in the accurate detection of myocardial involvement in SLE, and other rheumatological conditions, as it may potentially guide therapy aimed at reducing adverse cardiovascular outcomes.

A small study by Singh et al. [90] showed that T2 relaxation times were higher in six patients with active SLE compared to five with lower disease activity and five healthy controls (T2 values of 82, 64 and 65 ms respectively). Similarly, Abdel-Aty et al. [88] showed that both ER and gRE were significantly higher in patients with active disease, both correlated to disease activity and ER significantly decreased with clinical improvement.

Mavrogeni et al. [89] compared a group of twenty-five patients with active SLE and suspected cardiac involvement with fifty patients suspected of having viral myocarditis showing no statistical difference in ER and EGE, potentially suggesting similar myocardial pathological processes in both conditions.

Puntmann et al. [91] showed that T1 and ECV values were significantly higher among thirty-three SLE patients in clinical remission compared to twenty-one healthy controls (T1 1152 ± 46 vs. 1056 ± 27 ms, p < 0.001; ECV 30 ± 6% versus 26 ± 5%, p = 0.007). A challenge for the CMR community is to decipher whether such findings represent active inflammation or chronic fibrosis, or indeed both. The authors did not perform T2 mapping, however, ER did not differ between groups, potentially suggesting the T1 and ECV findings may represent fibrosis. Conversely, Zhang et al. [92] demonstrated higher T2 values in twenty-four SLE patients with low disease activity compared to twelve healthy controls (58.2 ± 5.6 vs. 52.8 ± 4.4 ms), which the authors suggested may represent ongoing myocardial inflammation.

LGE may be less prevalent in SLE. Zhang et al. [92] observed no late enhancement amongst twenty-four SLE patients while Mavrogeni et al. [89] found significantly less LGE amongst patients with active SLE compared to viral myocarditis (LGE volume 3.5 ± 5.5 vs. 8 ± 4.4%, p < 0.001), possibly reflecting a more diffuse nature of myocardial involvement.

## Rheumatoid arthritis

Rheumatoid arthritis (RA) is a chronic autoimmune disease [93]. Cardiovascular involvement is common, manifesting as coronary artery disease, myocardial inflammation and fibrosis, and is responsible for 40–80% of premature deaths [94–97].

Kobayashi et al. [98] examined eighteen RA patients without a previous history of cardiovascular conditions, finding LGE in almost 40% of patients, with a mostly non-ischaemic distribution. The presence of LGE was correlated to higher disease activity scores (DAS28 4.77 vs. 3.44, p = 0.011).

Mavrogeni et al. [99] used T2w imaging, EGE and LGE to compare two groups of RA patients in remission: twenty with and twenty without recent onset cardiac symptoms. 10% of patients with symptoms had evidence of myocardial infarction with a typical ischaemic LGE pattern and 65% displayed evidence of myocarditis as defined by LLC. Over three quarters of those diagnosed with myocarditis experienced an RA relapse within 6 weeks, possibly suggesting more active disease.

Ntusi et al. [100] found LGE to be present in almost half of twenty-eight examined RA patients with a mostly non-ischaemic, mid wall pattern. In addition, 5% of patients were diagnosed with silent myocardial infarction based on the presence of subendocardial LGE and confirmed by coronary angiography. There was no difference in global ER between RA patients and controls, however, RA patients had more areas of elevated ER (ER > 1.9, median 10 vs. 0% amongst controls) suggesting the presence of focal myocardial oedema. Finally, global T1 values and ECV were significantly higher in the RA group (T1 973 ± 27 vs. 961 ± 18 ms, p = 0.03; ECV 30.3 ± 3.4 vs. 27.9 ± 2%, p < 0.001). Although, in keeping with the findings in SLE, it is not clear to what extent these findings represent active inflammation or fibrosis and the magnitude of the difference in global T1, whilst statistically significant, were small.

It is clear from these CMR studies that subclinical cardiac involvement is common. CMR parameters have the potential to risk stratify and guide therapy in RA, although further work is required to define the nature of the CMR findings in RA and their accuracy and reproducibility in this population.

## Systemic sclerosis

Systemic Sclerosis (SSC) is an autoimmune connective tissue disorder characterised by multi-organ fibrosis [101]. Cardiac involvement in SSC is estimated at 15–35% [101] and includes myocardial fibrosis, myocarditis, dilated cardiomyopathy, premature coronary artery disease, conduction abnormalities, valvular and pericardial disease [102]. Myocardial pathologies are often subclinical with higher prevalence on autopsy studies [103]. Overt cardiac disease is associated with poor prognosis, with a reported 70% mortality at 5 years [104].

A number of studies have evaluated LGE in SSC patients, demonstrating a prevalence of LGE of between 4 and 66% [105–114]. LGE prevalence and distribution does not seem to differ between limited and diffuse cutaneous forms of SSC [105, 106, 108, 113]. Both non-ischaemic and ischaemic patterns of LGE are described [105–112] It is not clear whether the non-ischaemic LGE represents inflammation or fibrosis. Microvascular dysfunction is a prominent feature of SSC and diffuse myocardial ischaemia evident on perfusion imaging may be part of the pathophysiological process [107, 113].

In a study by Hachulla et al. [106] fifty-two SSC patients without prior cardiac disease were assessed by multiparametric CMR. Qualitative T2w signal was increased in 12% of participants. Ntusi et al. [110] study found nineteen SCC patients to have a significantly greater extent of high gRE values compared to twenty healthy controls [110]. There was no difference between limited and diffuse cutaneous SSC [106, 110].

T1 mapping and ECV values were also shown to be higher in SSC patients without past cardiovascular involvement. In previously mentioned study by Ntusi et al. [110], SSC participants had mean T1 values of 1007 ± 29 ms and ECV of 35.4 ± 4.8% compared to T1 of 958 ± 20 ms (p < 0.001) and ECV of 27.6 ± 2.5% (p < 0.001) amongst controls. Two further studies confirmed higher ECV in SSC patients compared to healthy controls: Barison et al. [109] (30 SSC patients, ECV 30 ± 4% vs. 28 ± 4%, p = 0.03) and Thuny et al. [115] (33 SSC patients, median ECV 30%, range 28–31.9% vs. 26.8%, range 25.4–29.1%, p = 0.001).

## Conclusions

By providing a ‘positive’ diagnostic test, CMR has changed the management of suspected viral myocarditis and has provided new insight into myocardial involvement in systemic inflammatory conditions. Thus CMR has opened a window for potential therapeutic targets. Parametric mapping appears to offer advantages over more conventional CMR techniques. However, multicentre, multivendor clinical trials are required to fully establish the clinical utility of CMR in myocarditis, and, in particular, quantitative mapping analysis.
